# How Biochar Affects Nitrogen Assimilation and Dynamics by Interacting Soil and Plant Enzymatic Activities: Quantitative Assessment of 2 Years Potted Study in a Rapeseed-Soil System

**DOI:** 10.3389/fpls.2022.853449

**Published:** 2022-03-10

**Authors:** Zaid Khan, Kangkang Zhang, Mohammad Nauman Khan, Junguo Bi, Kunmiao Zhu, Lijun Luo, Liyong Hu

**Affiliations:** ^1^MOA Key Laboratory of Crop Ecophysiology and Farming System in the Middle Reaches of the Yangtze River, College of Plant Science and Technology, Huazhong Agricultural University, Wuhan, China; ^2^Shanghai Agrobiological Gene Center, Shanghai, China

**Keywords:** biochar, nitrogen use efficiency, rapeseed, enzyme, nitrogen

## Abstract

The amendment of biochar has been proposed to improve soil fertility and crop yield. However, consolidated information lacks explaining the role of biochar on soil and plant enzymatic activities involved in nutrients cycling in soil and accumulation in plants improving utilization of applied inorganic fertilizer and crop growth. In the current study, we evaluated the integral effects of biochar levels (B0:0, B15:15, B3:30, and B60:60 t ha^–1^) and nitrogen fertilizer levels (N0:0, N75:75, N225:225, and N450:450 kg ha^–1^) on soil physicochemical properties, enzymatic activities, nitrogen use efficiency (NUE) and grain yield of rapeseed for 2 years in the pots during 2020 and 2021. The findings revealed that compared to control (B0 + N0), a combination of B30 + N450 increased soil urease activity by 73 and 75%, and B60 + N450 increased activities of soil catalase by 17 and 16%, and B60 + N225 increased alkaline phosphatase by 17 and 19%, respectively, in the first and second year. Moreover, a single application of high nitrogen at 450 kg ha^–1^ reduced the activities of plant nitrogen metabolism-related enzymes, however; the integration of biochar at 30 t ha^–1^ compensated the high nitrogen toxicity and improved the activities of nitrate reductase (NR), nitrite reductase NIR, glutamate synthase (GS) and glutamine synthetase (GOGAT) at seedling stage (SS) and flowering stage (FS) in both years. The integration of biochar at 30 t ha^–1^ with nitrogen at 450 kg ha^–1^ induced synergetic effects on rapeseed growth through sorption of excessive nitrogen in soil and significantly improved the plant height up to 11 and 18%, pods plant^–1^ 39 and 32% and grain yield plant^–1^ 54 and 64%, respectively, during the first and second year. Moreover, biochar at 15 t ha^–1^ along with nitrogen at 225 kg ha^–1^ resulted in the highest NUE of 29% in both years suggesting that biochar can also offset the deficiency of lower nitrogen. This study highlighted the ameliorative effect of biochar suppressing high nitrogen toxicity and decreasing lower nitrogen deficiency effects on rapeseed growth by improving nitrogen use efficiency via enhancing soil conditions, enzymatic activities and soil nitrogen utilization potential and thus improving rapeseed growth and yield.

## Introduction

In the recent past, global food production has greatly increased with applying chemical nitrogen (N) fertilizers. In China, a 70% increase in crop production has been recorded with a threefold increase in chemical N fertilizers since 1980 (Guo et al., 2010). However, application of N at such high levels will decrease nitrogen use efficiency as well as contaminate the environment due to ammonia volatilization, soil acidification, and water eutrophication (Guo et al., 2010; He et al., 2017). In addition, lower nitrogen use efficiency and poor soil fertility in many developing countries accelerate food insecurity to meet the food demand of the world’s growing population.

Rapeseed has been cultivating for thousands of years and producing edible and non-edible oil with 70.51 million t annual production of rapeseed yield (FAO, 2021). China has been one of the largest producers of rapeseed in the world since 1980 (Jones et al., 2013). However, compared to Australia, Germany, and other countries, China’s nitrogen fertilizer consumption is higher while the nitrogen agronomic efficiency is lower for rapeseed production (Li et al., 2016). The seed production of rapeseed per unit N applied is equal to the half of cereals, which ascribe it as a lower N-efficient crop (Sylvester-Bradley and Kindred, 2009). Therefore, it is imperative to apply nitrogen fertilizers along with organic amendments to prevent nitrogen fertilizer loss, lower the economic cost of production, alleviate environmental pollution and improve nitrogen use efficiency (NUE) and yield of rapeseed on a sustainable basis.

Biochar is prepared by pyrolysis of biomass in the absence of oxygen (Ahmad et al., 2014) and used as a soil organic amendment due to its crucial role in carbon sequestration and improving soil quality. The availability and uptake of plant nutrients are related to soil pH (Purakayastha et al., 2019), and organic matter and ash content in biochar can enhance soil pH and quality and consequently improve the utilization of nutrients. Furthermore, the organic matter portion of biochar contains nitrogen, and the ash portion contains phosphorus and potassium (Jindo et al., 2020), which indicates that biochar interacts with soil in many ways to optimize the concentration and uptake of these essential plant nutrients. The amendment of biochar having a porous structure and high surface area provides more space for the habitat of microbes (Pietikäinen et al., 2000), promoting the biochemical processes in the soil such as soil enzymatic activities that are involved in various nutrients cycling in the soil.

The soil factors, such as pH, soil organic matter, and water holding capacity (WHC), induce the activities of soil enzymes, and these soil factors are dependent on biochar addition depending on the nature of biochar, as biochar possess high retention ability and high concentrations of organic matter and ash (Ouyang et al., 2014). The biochar surface contains oxygen functional groups, which can adhere to more water particles that improve water holding capacity (WHC) and water use efficiency (WUE) of soil, consequently improving nutrients retention, soil fertility, and crop productivity (Rondon et al., 2007). Soil enzymes are critical members of soil, which help in decomposing soil organic matter (SOM) and nutrients translocation and regulate the variations in the soil conditions upon biochar addition (He et al., 2021). The soil catalase activity helps in synthesis of soil humus and abandon toxicity of hydrogen peroxide to soil enzymes (Bartkowiak and Lemanowicz, 2017) and invertase activity indicates the pattern of decomposition and accumulation of soil organic carbon for further utilization (Paz-Ferreiro et al., 2011). The concentrations of nitrogen and phosphorus in soil have a strong influence on the functions of urease and phosphatase (Oladele, 2019). The effect of biochar amendment on urease activity is more crucial as this enzyme plays a vital role in N cycling, availability, and N_2_O emissions in agricultural fields with substantial effects on the ecosystem (Harter et al., 2014). These promotions in soil conditions reflect the crucial role of biochar in altering the activities of soil enzymes that can also improve the mineralization and utilization of nitrogen through the activities of N assimilation enzymes such as nitrate reductase, nitrite reductase, glutamine synthetase, and glutamate synthase.

The preferable forms of plant nitrogen uptake through roots are nitrate-nitrogen (NO${}_{3}^{-}$-N) or ammonium-nitrogen (NH${}_{4}^{+}$-N) (Miller and Cramer, 2005). Once the NO${}_{3}^{-}$ is taken up by plants, it is reduced to nitrite (NO${}_{2}^{-}$) by nitrate reductase and then to ammonium ion (NH${}_{4}^{+}$) by nitrite reductase (Xiong et al., 2018). The ammonium is further incorporated by glutamine synthetase and glutamate synthase into glutamic acid, which supplies nitrogen to produce essential amino acids and proteins (Shah et al., 2017). Plant N content and concentrations of amino acids are primarily increased upon higher nitrogen fertilizer supply (Luo et al., 2008), but the effect of nitrogen use efficiency remained insignificant due to the hostile impacts of high nitrogen fertilization on soil and plant. The conventional application of high nitrogen fertilizers increases crop yields (Bitew and Alemayehu, 2017); however, half of the applied nitrogen fertilizers are lost into the environment (Lassaletta et al., 2014), and thus negatively affecting the environment, soil fertility, and nitrogen use efficiency. Previously, several strategies under biochar addition have been explained the retention and utilization of applied nitrogen fertilizers such as adsorption of N to biochar, high cation or anion reactions, and immobilization of N due to labile carbon of biochar (Clough and Condron, 2010).

In soil, the interaction between different fertilizers and nutrients occurs when the addition of one soil amendment affects the adsorption, availability, and utilization of other nutrients. Although many previous studies have investigated the effect of biochar and nitrogen fertilization, however; limited information is available to explain how biochar responds to high nitrogen fertilization and its consequences on rapeseed growth and production. In order to recognize the rapeseed growth under different nitrogen levels along with biochar, this study was designed to systematically and briefly examine the biochar effect on soil conditions, enzymatic activities (soil enzymes and plant N assimilation enzymes), physiological traits, growth, yield contents and seed quality of rapeseed.

## Materials and Methods

### Test Materials and Experimental Conditions

A 2 years’ outdoor experiment was conducted in plastic pots to investigate the integral effect of biochar and nitrogen on soil quality and morphology, physiology, and yield of rapeseed. After the rapeseed harvesting in the first year, direct-seeded rice was grown in the same pots and then followed by rapeseed for the second year experiment. Pored bottom plastic pots with 27 cm height and 25.5 and 22.5 cm above and below diameter were filled with sun-dried and sieved (2 mm) mixture of clay and sand (2:1) weighed 14 kg. Initially, five seeds of rapeseed cultivar Huayuoza 9 (HZ9, Hybrid) were sown and then thinned to three plants per pot after seedling establishment. Pots were irrigated with tap water throughout the growing period in both years to meet the irrigation water requirements. Proper agronomic management techniques were adapted at specific and required times of the trial duration.

### Experimental Design and Treatments Combination

The pot experiment was performed in a completely randomized design having four biochar levels (0, 15, 30, and 60 t ha^–1^) and four nitrogen levels (0, 75, 225, and 450 kg h^–1^). There was a total of 16 different treatments, and each treatment was repeated 15 times. Biochar at different levels equivalent to (B0: 0, B15: 105, B30: 210, and B60: 420 g per pot soil/weight ratio) was applied once at the start of the experiment and thoroughly mixed in the top 20 cm soil in the pot at the soil filling stage. Rice straw biochar was provided by Hubei Jinzhi Eco-Energy Co., Ltd, prepared by pyrolysis under a high temperature of 600°C (Table 1). Basic essential fertilizers (phosphorus and potassium) were applied as basal fertilizers at the time of sowing. Nitrogen was applied in two doses (50% at the time of sowing and 50% at stem elongation). A total amount of nitrogen applied as (N0: 0 g, N75: 1.14 g, N225: 3.42 g and N450: 6.84 g Urea) along with phosphorus (7.87 g P_2_O_5_) and potassium (1.83 g K_2_O).

**TABLE 1:** Chemical properties of biochar used and soil before the experiment.

Properties of biochar	Values	Properties of soil	Values
pH	9.42	pH	6.04
Nitrogen	0.74%	Electric conductivity	1.08
Carbon	47.14%	Total nitrogen	0.49 g kg^–1^
Hydrogen	1.62%	Total phosphorus	0.34 g kg^–1^
Oxygen	11.85%	Available potassium	76.24 mg kg^–1^
Potassium	18.9%	Soil organic carbon	4.62 g kg^–1^
Phosphorus	0.32%		
Ash	19.43%		

### Determination of Photosynthetic Processes

The measurement of photosynthesis (A) and transpiration (E) were performed at two different intervals seedling stage (SS) and flowering stage (FS) of rapeseed growth in both years. The fully expanded fourth flag leaf from bottom to top was selected each time by using a portable photosynthesis system (Li-6400, Li-COR Inc., Lincoln, NE, United States) during the daytime from 10:00 am to 03:00 pm in full sunshine under the following conditions; CO_2_-400 μmol mol^–1^, leaf temperature-23°C, light intensity-1,000 μmol m^–2^s^–1^, and air humidity-70%.

### Soluble Sugar and Crude Protein Contents Determination

Plant leaf samples were taken at seedling and flowering stages to measure the soluble sugar and crude protein contents in both years. The commercial kits of soluble sugar and crude protein (Suzhou Grace Biotechnology co., Ltd., Suzhou, China) were used, and absorbance was noted at 620 and 562 nm, respectively, to determine soluble sugar and crude protein contents expressed as mg g^–1^ FW.

### Determination of Plant Nitrogen Content, Total Nitrogen Accumulation, and Nitrogen Use Efficiency (NUE%)

Plant leaf samples were taken at the flowering stage for both years and oven-dried to measure nitrogen content using colorimetric analysis with a discrete analyzer (SmartChem 200, Unity Scientific, Brookfield, CT, United States) following Kjeldahl digestion. The following equations were used to determine the total nitrogen accumulation and nitrogen utilization efficiency.

TNA = N concentration × total plant dry weight (mg N) (Abenavoli et al., 2016).

NUE = N uptake in fertilized pots − N uptake in unfertilized pots × 100/N applied (%) (Khan et al., 2020).

### Determination of Plant and Soil Enzymatic Activities

Plant fresh leaf samples were taken at seedling and flowering stages in both years to estimate the activities of plant enzymes. The commercial kits of nitrate reductase NR, nitrite reductase NIR, glutamate synthase GS and glutamine synthetase GOGAT (Suzhou Grace Biotechnology co., Ltd., Suzhou, China) were used, and absorbance of 530, 540, 450, and 540 nm, respectively, was noted to determine their activities.

Similarly, fresh soil samples at the flowering stage were taken in both years to determine the activities of soil enzymes. The commercial kits of urease, soil catalase, alkaline phosphatase, and invertase (Suzhou Grace Biotechnology co., Ltd., Suzhou, China) were used, and absorbance of 578, 510, 405, and 540 nm, respectively, was noted to determine the activities of soil enzymes.

### Plant Growth and Yield Contents

Different plant growth and yield parameters (plant height, stem diameter, above-ground dry biomass, pods plant^–1^, grain yield plant^–1^, and thousand-grain weight) were measured after harvesting the plants of each pot.

### Soil Physiochemical Properties Analysis

Three soil samples were taken before the start of the experiment to measure the basic chemical properties of the trial soil. After harvesting rapeseed plants each year, three replicate soil samples from 20 cm top of the pot were taken and oven-dried for further investigation of the chemical properties of soil. 10 g dry soil samples were mixed at the ratio of 1:2 in distilled water and mixed properly by shaking at 180 rounds/minute for 20 min to measure soil pH and EC by digital pH meter (B-212, HORIBA, Ltd., Kyoto City, Japan) and EC meter (B-173, HORIBA, Ltd., Kyoto City, Japan). Colorimetric analysis was performed using a discrete analyzer to measure soil total phosphorus TP content after Kjeldahl digestion (SmartChem 200, Unity Scientific, Brookfield, CT, United States). Soil available potassium AK was measured with a flame photometer after Kjeldahl digestion. Indophenol methods (Kovar and Pierzynski, 2009) and the Cataldo method (Cataldo et al., 1974) were used to measure NH${}_{4}^{+}$-N and NO${}_{3}^{-}$-N, respectively. 0.3 g soil was weighed to measure the soil organic carbon with wet digestion according to the standard protocol (Cataldo et al., 1975).

The soil samples were taken each year after the harvesting of rapeseed to measure the soil physical properties of each treatment. The core method was followed to measure soil bulk density BD (Grossman and Reinsch, 2002). Furthermore, the obtained soil BD value was used by following the equation (Hillel, 2004) to calculate soil total porosity. Similarly, the soil moisture content was measured by following the method (Ledieu et al., 1986).

### Statistical Analysis

Two-way analysis of variance (ANOVA) was performed to determine significant differences between different treatments by analyzing four replicates of each treatment with statistix 8.1 (*p* < 0.05). The individual means of different treatments were compared by LSD test. Figures were drawn using OriginPro 2018. Correlation coefficients principal component analysis (PCA) of soil physiochemical properties, enzymes, and yield were drawn with Pearson correlation analysis using R studio version 1.1.

## Results

### Biochar Effect on Physiochemical Properties of Post-harvest Soil

The soil physiochemical properties differed along with different biochar levels in both consecutive years, indicating the positive role of biochar, while the nitrogen fertilization effect was less significant than biochar (Tables 2, 3). In the range of 0–60 t ha^–1^ biochar application, the results showed that soil chemical and physical properties were improved more significantly under biochar application at 30–60 t ha^–1^ in the first season; however, in the second season, the higher biochar at 60 t ha^–1^ reduced the availability of available forms of nitrogen (NO${}_{3}^{-}$ and NH${}_{4}^{+}$) compared to pots amended with biochar 30 t ha^–1^. In the first season of rapeseed, biochar application treatment B60 + N225 significantly increased soil pH by 12%, soil organic carbon (SOC) by 359% in B60 + N225, NO${}_{3}^{-}$ by 198% in B30 + N450, NH${}_{4}^{+}$ by 134% in B60 + N450, TP by 68% in B60 + N450 and AK by 64% in B60 + N225 treatment compared to control (B0 + N0) (Table 2). Similarly, in the second season under rapeseed cultivation, the combined application of biochar and nitrogen fertilizer significantly increased soil pH by 8% in B60 + N225, SOC by 308% in B60 + N450, NO${}_{3}^{-}$ by 139% in B30 + N450, NH${}_{4}^{+}$ by 159% in B30 + N450, TP by 61% in B60 + N450 and AK by 65% in B60 + N75 treatment compared to control (Table 3). The interaction between biochar and nitrogen was found significant for NO${}_{3}^{-}$ and NH${}_{4}^{+}$ in both years, indicating the positive role of biochar in improving nitrogen fertilizer utilization by increasing the concentrations of available nitrogen for plant uptake.

**TABLE 2:** Integrated effect of biochar and nitrogen application on chemical properties of soil during 2019–2020.

Biochar	Nitrogen	Soil pH	SOC (g kg^–1^)	NO${}_{3}^{-}$-N (mg kg^–1^)	NH${}_{4}^{+}$-N (mg kg^–1^)	TP (g kg^–1^)	AK (mg kg^–1^)
B0	N0	6.37 f	3.89 e	9.54 h	4.48 gh	0.32 k	109.24 f
	N75	6.46 e	4.06 e	12.44 fg	4.81 fgh	0.33 jk	107.90 f
	N225	6.43 ef	3.92 e	17.66 d	5.76 defg	0.38 hij	111.48 f
	N450	6.41 ef	4.11 e	21.04 c	6.09 def	0.38 hij	109.93 f
B15	N0	6.74 cd	6.84 d	13.54 efg	4.97 efgh	0.36 ij	128.10 e
	N75	6.67 d	7.05 d	18.16 d	6.50 de	0.39 ghi	127.05 e
	N225	6.75 c	7.25 d	24.54 b	6.25 def	0.40 fgh	129.96 e
	N450	6.69 cd	7.15 d	25.11 b	8.76 b	0.42 fg	128.39 e
B30	N0	6.91 b	11.39 c	13.66 efg	4.19 gh	0.42 fg	156.36 d
	N75	6.89 b	11.38 c	15.70 de	6.20 def	0.44 ef	162.28 c
	N225	6.86 b	12.49 c	24.21 b	6.80 cd	0.46 de	158.20 cd
	N450	6.87 b	11.35 c	27.74 a	10.49 a	0.50 bc	159.12 cd
B60	N0	7.14 a	16.02 b	11.70 gh	4.01 h	0.46 de	170.26 b
	N75	7.13 a	16.62 b	14.91 ef	5.42 defgh	0.47 cd	176.19 a
	N225	7.16 a	17.88 a	23.22 bc	6.19 def	0.52 ab	179.35 a
	N450	7.09 a	16.08 b	28.44 a	8.34 bc	0.54 a	176.83 a
***F*-values and significance level**							
Biochar		553.67***	685.67***	32.92***	6.99*	153.68***	1065.81***
Nitrogen		1.54 ns	2.16 ns	206.77***	37.06***	33.54***	1.99 ns
Biochar × Nitrogen		1.64 ns	1.01 ns	2.79*	2.36*	0.76 ns	1.48 ns

B0, 0 t ha^–1^ biochar; B15, 15 t ha^–1^ biochar; B30, 30 t ha^–1^ biochar; B60, 60 t ha^–1^ biochar; N0, 0 kg ha^–1^ nitrogen; N150, 150 kg ha^–1^ nitrogen; N300, 300 kg ha^–1^ nitrogen, and N450, 450 kg ha^–1^ nitrogen. SOC, soil organic carbon; NO${}_{3}^{-}$-N, nitrate nitrogen; NH${}_{4}^{+}$-N, ammonium nitrogen; TP, total phosphorus; and AK, available potassium. Different letters within the columns show a significant difference between the treatments at p = 0.05 according to the LSD test, and asterisks represent a significant difference at *p < 0.05, **p < 0.01, ***p < 0.001 level; ns, not-significant.

**TABLE 3:** Integrated effect of biochar and nitrogen application on chemical properties of soil during 2020–2021.

Biochar	Nitrogen	Soil pH	SOC (g kg^–1^)	NO_3_^–^ (mg kg^–1^)	NH${}_{4}^{+}$ (mg kg^–1^)	TP (g kg^–1^)	AK (mg kg^–1^)
B0	N0	6.79 g	4.78 e	14.24 i	5.68 h	0.36	114.56 f
	N75	6.80 g	4.67 e	14.56 i	6.21 gh	0.40 g	118.76 f
	N225	6.88 f	4.60 e	18.69 gh	8.53 def	0.40 g	116.66 f
	N450	6.82 g	4.49 e	26.41 d	9.58 cd	0.44 ef	114.97 f
B15	N0	7.24 e	9.19 d	16.58 hi	6.73 gh	0.42 fg	141.67 de
	N75	7.25 de	10.11 d	19.53 fg	7.12 fgh	0.42 fg	136.85 e
	N225	7.27 de	9.15 d	26.91 cd	8.94 cde	0.45 ef	142.95 d
	N450	7.24 e	10.46 d	31.15 b	12.28 b	0.46 e	143.96 d
B30	N0	7.29 bcde	13.53 c	16.36 hi	6.01 h	0.47 e	166.10 c
	N75	7.30 bcd	13.83 c	22.41 e	7.05 fgh	0.50 d	172.42 b
	N225	7.29 bcde	13.51 c	26.58 d	10.35 c	0.54 b	169.52 bc
	N450	7.28 cde	14.48 c	34.11 a	14.75 a	0.54 b	167.36 bc
B60	N0	7.34 ab	17.75 b	15.74 i	5.67 h	0.51 bcd	185.54 a
	N75	7.33 abc	18.23 ab	21.95 ef	6.98 gh	0.53 bc	190.05 a
	N225	7.36 a	19.52 a	24.59 de	7.63 efg	0.57 a	188.33 a
	N450	7.34 ab	19.46 a	29.48 bc	12.06 b	0.58 a	186.15 a
***F*-values and significance level**							
Biochar		568.52***	531.67***	33.60***	11.43**	199.14***	977.74***
Nitrogen		2.74 ns	2.06 ns	171.41***	110.56***	35.84***	1.43 ns
Biochar × Nitrogen	1.00 ns	1.09 ns	2.97*	3.79*	1.52 ns	1.65 ns	

B0, 0 t ha^–1^ biochar; B15, 15 t ha^–1^ biochar; B30, 30 t ha^–1^ biochar; B60, 60 t ha^–1^ biochar; N0, 0 kg ha^–1^ nitrogen; N150, 150 kg ha^–1^ nitrogen; N300, 300 kg ha^–1^ nitrogen, and N450, 450 kg ha^–1^ nitrogen. SOC, soil organic carbon; NO${}_{3}^{-}$-N, nitrate nitrogen; NH${}_{4}^{+}$-N, ammonium nitrogen; TP, total phosphorus and AK, available potassium. Different letters within the columns show a significant difference between the treatments at p = 0.05 according to the LSD test, and asterisks represent a significant difference at *p < 0.05, **p < 0.01, and ***p < 0.001 level; ns, not-significant.

The findings of both seasons under rapeseed cultivation revealed that soil physical properties significantly responded to biochar amendment; however, only soil total porosity responded significantly to nitrogen fertilizer (Table 4). In the first year, the soil total porosity was 39.74% in control and 51.87% in B60 + N450 treatment, soil bulk density ranged from 1.12 g cm^–3^ in control to 1.05 g cm^–3^ in B60 + N225 treatment and soil moisture content 8.96% in control and 14.63% in B60 + N225 treatment (Table 4). Likewise, in the second year, the soil total porosity increased from 41.99% in control to 56.38% in B60 + N450 treatment, soil bulk density decreased from 1.08 g cm^–3^ in control to 1.02 g cm^–3^ in B60 + N450 treatment and soil moisture from 11.17% in control to 14.83% in B60 + N0 treatment (Table 4).

**TABLE 4:** Integrated effect of biochar and nitrogen application on physical properties of soil during 2019–2020 and 2020–2021.

Biochar	Nitrogen	Total soil porosity (%)		Soil bulk density (g cm^–3^)		Soil moisture content (%)	
		2019–2020	2020–2021	2019–2020	2020–2021	2019–2020	2020–2021
B0	N0	39.74 j	41.99 h	1.12 a	1.08 ab	8.96 g	11.17 e
	N75	40.50 ij	42.11 h	1.11 a	1.10 a	9.51 fg	11.13 e
	N225	43.18 ghi	44.01 gh	1.11 a	1.09 a	8.57 g	11.80 cde
	N450	43.15 ghi	45.90 fg	1.08 bc	1.08 ab	10.05 efg	11.57 de
B15	N0	42.57 hij	46.74 ef	1.07 bcd	1.07 bcd	10.83 def	12.82 abcde
	N75	44.84 fgh	46.82 ef	1.08 b	1.06 cde	11.96 bcd	13.74 abc
	N225	45.40 efgh	49.06 de	1.08 bc	1.07 bcd	12.90 abc	12.53 bcde
	N450	45.77 defg	48.78 de	1.07 bcd	1.05 def	11.66 cde	12.87 abcde
B30	N0	45.36 ghi	48.94 de	1.06 cde	1.06 cde	11.86 bcde	12.22 bcde
	N75	47.25 cdef	50.91 cd	1.06 de	1.05 defg	12.90 abc	13.93 abc
	N225	47.01 cdef	50.51 cd	1.07 bcde	1.03 gh	13.29 abc	12.54 bcde
	N450	47.92 cde	51.92 bc	1.06 de	1.03 gh	12.87 abc	13.07 abcde
B60	N0	48.62 bcd	54.21 ab	1.06 de	1.03 gh	13.39 abc	14.83 a
	N75	49.02 abc	54.23 ab	1.06 de	1.03 gh	13.67 ab	13.78 abc
	N225	51.09 ab	55.30 a	1.05 e	1.03 gh	12.07 bcd	14.18 ab
	N450	51.87 a	56.38 a	1.06 de	1.02 h	14.63 a	13.38 abcd
***F*-values and significance level**							
Biochar		52.06***	116.54***	39.10***	49.73***	32.97***	8.53**
Nitrogen		7.81**	7.70**	1.87 ns	2.63 ns	1.98 ns	0.28 ns
Biochar × Nitrogen		0.40 ns	0.58 ns	1.66 ns	1.32 ns	1.53 ns	0.71 ns

B0, 0 t ha^–1^ biochar; B15, 15 t ha^–1^ biochar; B30, 30 t ha^–1^ biochar; B60, 60 t ha^–1^ biochar; N0, 0 kg ha^–1^ nitrogen; N150, 150 kg ha^–1^ nitrogen; N300, 300 kg ha^–1^ nitrogen, and N450, 450 kg ha^–1^ nitrogen. Different letters within the columns show a significant difference between the treatments at p = 0.05 according to the LSD test, and asterisks represent a significant difference at *p < 0.05, **p < 0.01, and ***p < 0.001 level; ns, not-significant.

### Soil Enzymatic Activities

**FIGURE 1:**
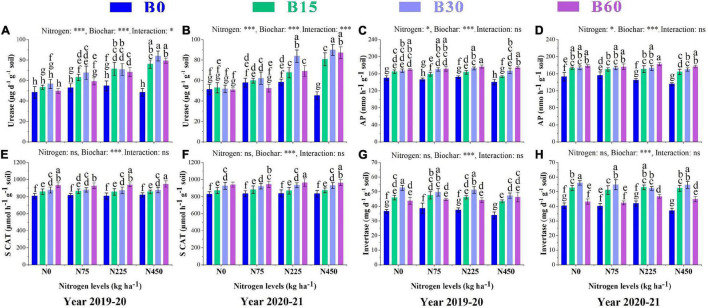
Integral effects of biochar and nitrogen on soil urease **(A)** 2019–2020, **(B)** 2020–2021, alkaline phosphatase **(C)** 2019–2020, **(D)** 2020–2021, soil catalase **(E)** 2019–2020, **(F)** 2020–2021 and invertase **(G)** 2019–2020 and **(H)** 2020–2021. B0: 0 t ha^−1^biochar; B15: 15 t ha^−1^biochar; B30: 30 t ha^−1^biochar; B60: 60 t ha^−1^biochar; N0: 0 kg ha^−1^ nitrogen; N75: 75 kg ha^−1^ nitrogen; N225: 225 kg ha^−1^ nitrogen, and N450: 450 kg ha^−1^ nitrogen, error bars represent standard error for each treatment in three replicates. Different letters on each bar represent significant difference according to LSD test (*p* < 0.05).

Soil enzymes are considered strong indicators of soil status and fertility as their activities are related to soil type, quality, and physiochemical properties. The significant changes in urease, soil catalase, alkaline phosphatase, and invertase caused by the combined application of biochar and nitrogen are shown in Figure 1. The urease activity was higher in the pots treated with B30 + N450 with an increase of 73 and 75%, respectively, in the first and second year than that in the control treatment (Figures 1A,B). The activity of alkaline phosphatase increased by 10% in B15 + N0, 15% in B30 + N225, and 17% in B60 + N225 in the first year, and 11% in B15 + N75, 13% in B30 + N75, and 19% in B60 + N225 in the second year as compared to control treatment (Figures 1C,D). The nitrogen fertilizer effect was not significant, and biochar amendment significantly improved the activity of soil catalase by 7% in B15 + N75, 8% in B30 + N75 and 17% in B60 + N450 in the first year and 6% in B15 + N75, 12% in B30 + N225, and 16% in B60 + N225 treatment compared to control in the second year (Figures 1E,F). The activity of invertase responded significantly only to biochar addition and increased by 29% in B15 + N75, 44% in B30 + N0 and 26% in B60 + N450 in the first year and 31% in B15 + N225, 38% in B30 + N0, and 15% in B60 + N225 in the second year as compared to control treatment (Figures 1G,H).

### Activities of Plant Nitrogen Metabolism Associated Enzymes

**FIGURE 2:**
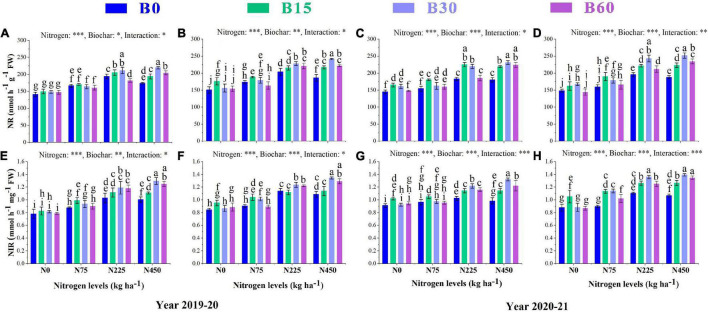
Integral effects of biochar and nitrogen on nitrate reductase NR **(A)** SS, **(B)** FS in 2019–2020 and **(C)** SS, **(D)** FS in 2020–2021 and nitrite reductase **(E)** SS, **(F)** FS in 2019–2020 and **(G)** SS, **(H)** FS in 2020–2021. SS: seedling stage, FS: flowering stage. B0: 0 t ha^−1^biochar; B15: 15 t ha^−1^biochar; B30: 30 t ha^−1^biochar; B60: 60 t ha^−1^biochar; N0: 0 kg ha^−1^ nitrogen; N75: 75 kg ha^−1^ nitrogen; N225: 225 kg ha^−1^ nitrogen, and N450: 450 kg ha^−1^ nitrogen, error bars represent standard error for each treatment in three replicates. Different letters on each bar represent significant differences according to the LSD test (*p* < 0.05).

**FIGURE 3:**
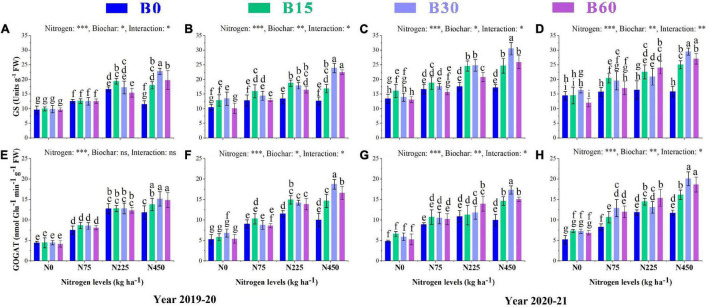
Integral effects of biochar and nitrogen on glutamate synthase GS **(A)** SS and **(B)** FS in 2019–2020 and **(C)** SS and **(D)** FS in 2020–2021 and glutamine synthetaseGOGAT **(E)** SS and **(F)** FS in 2019–2020 and **(G)** SS and **(H)** FS in 2020–2021. SS: seedling stage, FS: flowering stage. B0: 0 t ha^−1^biochar; B15: 15 t ha^−1^biochar; B30: 30 t ha^−1^biochar; B60: 60 t ha^−1^biochar; N0: 0 kg ha^−1^ nitrogen; N75: 75 kg ha^−1^ nitrogen; N225: 225 kg ha^−1^ nitrogen, and N450: 450 kg ha^−1^ nitrogen, error bars represent standard error for each treatment in three replicates. Different letters on each bar represent significant differences according to the LSD test (*p* < 0.05).

The growth and development of a plant are primarily dependent on nitrogen, an essential and limiting nutrient in the soil. Its uptake and assimilation occur through the activities of various nitrogen-related enzymes. The results showed a significant variation in activities of nitrate reductase NR, nitrite reductase NIR, glutamate synthase GS and glutamine synthetase GOGAT caused by the integral effect of biochar and nitrogen fertilizer (Figures 2, 3). The sole addition of nitrogen fertilizer (B0 + N225) significantly increased the activities of N enzymes as compared to control (B0 + N0); however, the single application of high nitrogen (B0 + N450) compared to (B0 + N225) negatively affected the enzymatic activities and reduced NR by 10 and 8%, NIR by 2 and 4%, GS by 30 and 5% and GOGAT by 7 and 13% in the first year and NR by1 and 3%, NIR by 4 and 3%, GS by 2 and 3%, GOGAT by 8 and 1% in the second year, respectively, at seedling stage (SS) and flowering stage (FS) (Figures 2, 3).

The findings of our study revealed a significant interaction between different combinations of biochar and nitrogen levels which alleviated the adverse effects of high nitrogen on the activities of N-related enzymes. Among different biochar levels, the combination of biochar and nitrogen (B30 + N450) significantly increased the activities of NR by 26 and 29%, NIR by 28 and 23%, GS by 97 and 89%, and GOGAT by 28 and 87% in the first year and NR by 28 and 33%, NIR by 34 and 30%, GS by 77 and 86%, GOGAT by 74 and 71% in the second year, respectively, at SS and FS as compared to (B0 + N450) (Figures 2, 3).

### Plant N Content, Total Nitrogen Accumulation, and Nitrogen Use Efficiency

**FIGURE 4:**
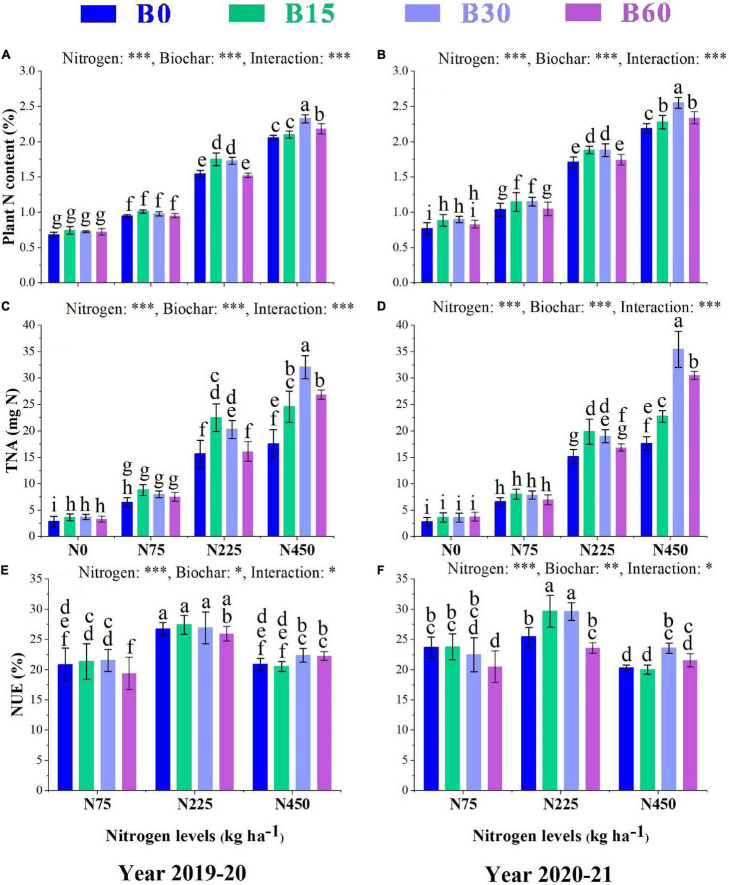
Integral effects of biochar and nitrogen on plant N content **(A)** 2019–2020 and **(B)** 2020–2021, total nitrogen accumulation TNA **(C)** 2019–2020 and **(D)** 2020–2021 and nitrogen use efficiency **(E)** 2019–2020 and **(F)** 2020–2021. B0: 0 t ha^−1^biochar; B15: 15 t ha^−1^biochar; B30: 30 t ha^−1^biochar; B60: 60 t ha^−1^biochar; N0: 0 kg ha^−1^ nitrogen; N75: 75 kg ha^−1^ nitrogen; N225: 225 kg ha^−1^ nitrogen, and N450: 450 kg ha^−1^ nitrogen, error bars represent standard error for each treatment in three replicates. Different letters on each bar represent significant differences according to the LSD test (*p* < 0.05).

The positive effect of biochar on nitrogen application promoted the soil physiochemical properties, enzymatic activities, and plant N-related enzymes dynamics and thus significantly enhanced the plant N content, TNA, and NUE (Figure 4). The increment in nitrogen fertilizer levels increased N content by 201 and 183% in B0 + N450 compared to B0 + N0 treatment, respectively, in the first and second year, while the addition of biochar along with nitrogen increased the N content by 12 and 16% in B30 + N450 compared to B0 + N450 treatment, respectively, in the first and second year (Figures 4A,B). Similarly, TNA increased by 515 and 524% in B0 + N450 compared to B0 + N0 treatment, respectively, in the first and second year and combined application of biochar and nitrogen increased TNA by 104 and 100% in B30 + N450, respectively, in both years compared to high sole application of nitrogen B0 + N450 (Figures 4C,D). Among all the treatments, the combination of B15 + N225 yielded the highest NUE with an increase of 16 and 6%, respectively, in the first and second year as compared to the high sole application of nitrogen fertilizer B0 + N450 (Figures 4E,F), which indicates the positive and synergetic role of biochar in elevating the uptake and utilization of nitrogen fertilizer.

### Biochar Effect on Soluble Sugar and Crude Protein Contents

**FIGURE 5:**
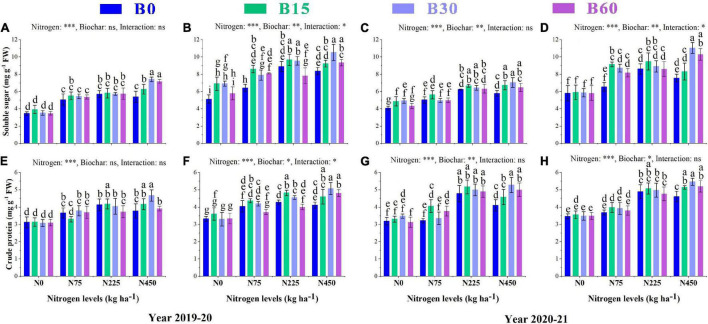
Integral effects of biochar and nitrogen on soluble sugar **(A)** SS and **(B)** FS in 2019–2020 and **(C)** SS and **(D)** FS in 2020–2021 and crude protein **(E)** SS and **(F)** FS in 2019–2020 and **(G)** SS and **(H)** FS in 2020–2021. SS: seedling stage, FS: flowering stage. B0: 0 t ha^−1^biochar; B15: 15 t ha^−1^biochar; B30: 30 t ha^−1^biochar; B60: 60 t ha^−1^biochar; N0: 0 kg ha^−1^ nitrogen; N75: 75 kg ha^−1^ nitrogen; N225: 225 kg ha^−1^ nitrogen, and N450: 450 kg ha^−1^ nitrogen, error bars represent standard error for each treatment in three replicates. Different letters on each bar represent significant differences according to the LSD test (*p* < 0.05).

The findings of the current study revealed that total soluble sugar increased with increasing nitrogen single application levels from 0 to 225 kg ha^–1^ and declined at 450 kg ha^–1^ compared to 225 kg ha^–1^ by 5% and 6% during the first year and 7 and 12% during the second year, respectively, at seedling and flowering stages due to the negative effect of high nitrogen fertilizer (Figures 5A–D). The total soluble sugar content in rapeseed leaves was significantly induced by ameliorating effect of biochar and nitrogen integration with B30 + N450 showing much higher total soluble sugar content among all other treatments with an increase of 113 and 105% in the first year and 72 and 89% in the second year, respectively, at seedling and flowering stages as compared to B0 + N0 treatment (Figures 5A–D). There were also significant interactions found between biochar and nitrogen at the flowering stage in the first year and both stages in the second year as total soluble sugar increased with increasing biochar application rate from 0 to 30 t ha^–1^ while the effect of 60 t ha^–1^ was less effective.

The concentration of crude protein responded significantly to sole application of nitrogen fertilizer, however; the content of crude protein at a high level of sole nitrogen B0 + N450 decreased by 8 and 4% at the first year and 14 and 5% at second year, respectively at both stages as compared to B0 + N225 (Figures 5E–H). The combined application of biochar and nitrogen B30 + N450 significantly reduced the adverse effect of high nitrogen and was found to be a superior treatment by increasing the crude protein content up to 49 and 52% during the first season and 65 and 57% during the second season, respectively, at both growth stages (Figures 5E–H).

### Photosynthetic Traits

The plant physiological processes are closely related to moisture availability and nutrient concentration in the soil. The effect of biochar was not significant during the seedling stage in the first year, and the high nitrogen toxicity of B0 + N450 negatively affected photosynthesis by 21% and transpiration by 15% as compared to normal nitrogen B0 + N225 (Table 5). Interestingly, the amendment of biochar at 30 t ha^–1^ along with nitrogen at 450 kg ha^–1^ positively improved the soil status and decreased the nitrogen adversity at the flowering stage by enhancing the photosynthesis rate up to 46% and transpiration 37% as compared to high sole nitrogen B0 + N450 treatment (Table 5). In comparison to B0 + N225 treatment, the application of high sole nitrogen B0 + N450 reduced the rates of photosynthesis up to 12 and 7% and transpiration 18% at seedling and flowering stages during the second year (Table 5). However, the response of biochar was found to be significant to reduce the high nitrogen toxicity in both stages, and combination of B30 + N450 improved the photosynthesis rate by 64 and 29% and transpiration by 52 and 23%, respectively, at seedling and flowering stages in the second year (Table 5). These results indicate the positive role of biochar in alleviating the adverse effects of high nitrogen on plant physiological processes by adsorbing the excess nitrogen from the soil to reduce nitrogen toxicity and releasing the stored nitrogen to reduce nitrogen deficiency.

**TABLE 5:** Integrated effect of biochar and nitrogen application on rapeseed photosynthesis and transpiration rates during 2019–2020 and 2020–2021.

Biochar	Nitrogen	Photosynthesis (umol CO_2_ m^–2^)				Transpiration (mmol H_2_O m^–2^ s^–1^)			
		2019–2020		2020–2021		2019–2020		2020–2021	
		Seedling	Flowering	Seedling	Flowering	Seedling	Flowering	Seedling	Flowering
B0	N0	7.39 g	15.15 h	8.67 i	16.27 f	2.47 cd	3.08 g	3.33 g	4.06 ef
	N75	9.27 ef	16.84 gh	12.69 fg	20.60 e	2.52 cd	3.85 efg	3.51 fg	4.52 cdef
	N225	16.34 a	22.04 e	18.23 cd	26.67 bcd	3.60 ab	4.78 de	4.70 bcde	6.23 ab
	N450	12.76 d	19.41 f	15.93 de	24.72 d	3.04 bc	4.39 ef	3.82 efg	5.49 abcde
B15	N0	7.59 fg	16.47 gh	11.33 gh	19.08 ef	2.53 cd	3.81 efg	3.73 efg	5.58 abcd
	N75	9.62 e	21.86 e	15.04 ef	21.18 e	2.70 cd	4.75 de	4.34 cdef	5.89 abc
	N225	16.55 a	25.88 bc	22.07 b	27.27 bcd	3.87 a	5.93 ab	4.95 abcd	6.58 a
	N450	14.00 cd	23.78 cde	22.63 b	29.87 abc	3.50 ab	4.85 bcde	5.04 abc	6.68 a
B30	N0	8.24 efg	16.65 gh	10.84 ghi	18.07 ef	2.48 de	3.77 efg	3.76 efg	4.75 bcdef
	N75	8.55 efg	17.83 fg	15.01 ef	24.75 d	2.50 cd	4.33 ef	3.98 defg	5.70 abc
	N225	16.09 ab	24.75 bcd	25.93 a	28.86 abc	4.04 a	5.60 abcd	5.79 a	5.86 abc
	N450	14.39 bcd	28.35 a	26.21 a	31.97 a	3.92 a	6.03 a	5.81 a	6.80 a
B60	N0	7.37 g	16.00 gh	9.52 hi	18.73 ef	2.27 d	3.45 fg	3.37 fg	3.39 f
	N75	8.66 efg	15.87 gh	10.73 ghi	20.85 e	2.30 d	3.95 efg	3.54 fg	4.01 ef
	N225	15.74 abc	23.60 de	18.79 c	26.36 cd	3.66 ab	4.82 cde	5.08 abc	4.15 def
	N450	15.12 abc	26.50 ab	23.90 ab	29.15 ab	3.78 a	5.89 abc	5.34 ab	4.90 bcde
***F*-values and significance level**									
Biochar		0.44 ns	19.76***	28.94***	7.54**	1.55 ns	4.70**	5.83**	12.38***
Nitrogen		159.74***	123.75***	170.82***	75.08***	35.23***	21.00***	21.79***	7.08**
Biochar × Nitrogen		1.09 ns	7.81**	5.70**	1.45 ns	0.86 ns	1.36 ns	1.29 ns	0.55 ns

B0, 0 t ha^–1^ biochar; B15, 15 t ha^–1^ biochar; B30, 30 t ha^–1^ biochar; B60, 60 t ha^–1^ biochar; N0, 0 kg ha^–1^ nitrogen; N150, 150 kg ha^–1^ nitrogen; N300, 300 kg ha^–1^ nitrogen, and N450, 450 kg ha^–1^ nitrogen. Different letters within the columns show a significant difference between the treatments at p = 0.05 according to the LSD test, and asterisks represent a significant difference at *p < 0.05, **p < 0.01, and ***p < 0.001 level; ns, not-significant.

### Biochar Effect on Growth, Yield, and Seed Quality of Rapeseed

**FIGURE 6:**
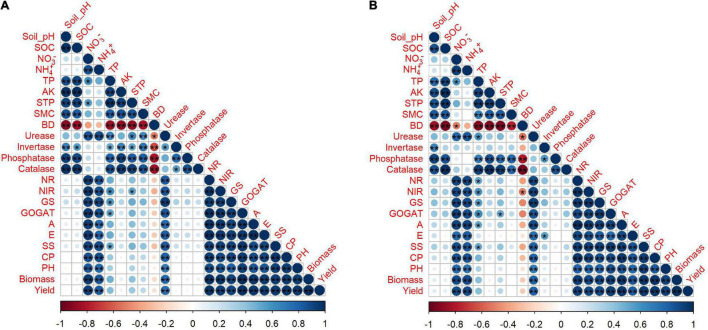
Correlation coefficients between soil physiochemical properties, growth and yield traits in 2019–2020 **(A)** and 2020–2021 **(B)** and asterisks represent a significant difference at *p < 0.05, ^**^p < 0.01. SOC: soil organic carbon, TP: total phosphorus, AK: available potassium, STP: soil total porosity, SMC: soil moisture content, BD: bulk density, NR: nitrate reductase, NIR: nitrite reductase, GS: glutamate synthase, GOGAT: glutamine synthetase, A: photosynthesis, E: transpiration, SS: soluble sugar, CP: crude protein, PH: plant height.

The plant growth, biomass and yield are the end products of a crop which reflects the capability of soil and productivity of a plant under certain conditions (Figure 6). The increase in biochar level from 0 to 30 t ha^–1^ and sole application of nitrogen from 0 to 225 kg ha^–1^ improved rapeseed growth while the biochar at 60 t ha^–1^ and nitrogen at 450 kg ha^–1^ adversely affected the rapeseed growth and yield (Table 6). The addition of high nitrogen B0 + N450 compared to B0 + N225 significantly reduced rapeseed plant height up to 3 and 5%, aboveground dry biomass 12 and 13%, pods plant^–1^ 7 and 6% and grain yield plant^–1^ 8 and 17%, respectively, during the first and second year (Table 6). However, the ameliorative role of biochar at 30 t ha^–1^ coupled with nitrogen at 450 kg ha^–1^ induced significant effects on rapeseed growth by absorbing the extra and excessive nitrogen and increased the plant height up to 11 and 18%, total biomass 65 and 72%, pods plant^–1^ 39 and 32% and grain yield plant^–1^ 54 and 64%, respectively, during the first and second year (Table 6).

**TABLE 6:** Integrated effect of biochar and nitrogen application on rapeseed growth and yield contents during 2019–2020 and 2020–2021.

Biochar	Nitrogen	Plant height (cm)		Aboveground dry biomass (g)		Pods plant^–1^		Grain yield plant^–1^ (g)		1,000 grain weight (g)	
						
		2019–2020	2020–2021	2019–2020	2020–2021	2019–2020	2020–2021	2019–2020	2020–2021	2019–2020	2020–2021
B0	N0	86.31 g	89.98 g	5.40 f	7.97 h	32.67 j	37.67 g	2.13 g	2.33 f	2.48 bcd	2.51 e
	N75	139.23 ef	143.00 e	27.84 e	28.89 fg	139.67 i	143.67 f	9.99 ef	10.65 e	2.50 abcd	2.86 abcde
	N225	156.13 bcd	153.49 cd	53.71 cd	58.54 cd	274.00 ef	292.67 de	16.79 cd	18.98 c	2.44 bcd	2.62 cde
	N450	150.61 cd	145.19 e	46.87 d	50.54 e	253.33 fg	272.33 e	15.30 d	15.74 d	2.43 cd	3.17 a
B15	N0	89.77 g	92.15 g	6.90 f	8.84 h	41.67 j	48.33 g	2.30 g	3.07 f	2.49 abcd	2.64 cde
	N75	146.19	156.23 bc	32.89 e	35.76 f	176.33 h	163.00 f	11.77 e	11.52 e	2.57 abc	3.01 abc
	N225	158.34 abc	163.67 ab	61.07 bc	66.30 bc	303.67 cd	314.33 cd	21.03 ab	19.07 c	2.35 d	2.73 bcde
	N450	160.88 abc	158.70 bc	61.03 bc	59.17 cd	317.00 bc	324.33 bc	17.47 cd	21.67 b	2.55 abc	2.89 abcde
B30	N0	84.39 g	87.50 g	6.09 f	6.04 h	38.00 j	44.33 g	2.26 g	2.70 f	2.48 bcd	2.72 bcde
	N75	136.62	141.69 e	25.13 e	29.40 fg	163.33 h	159.00 f	9.30 ef	10.09 e	2.56 abc	2.73 bcde
	N225	157.81 abc	154.89 c	58.79 bc	61.35 c	293.33 de	307.00 cd	18.94 bc	18.39 c	2.45 bcd	3.14 a
	N450	168.04 a	171.60 a	77.43 a	87.39 a	352.33 a	360.67 a	23.68 a	25.94 a	2.44 bcd	3.04 ab
B60	N0	80.63 g	88.14 g	6.35 f	5.06 h	36.33 j	41.00 g	2.22 g	2.26 f	2.44 cd	2.62 cde
	N75	129.00 f	132.12 f	22.75 e	25.93 g	168.67 h	149.33 f	8.72 f	9.72 e	2.52 abc	2.84 abcde
	N225	146.40 de	145.48 de	55.00 cd	53.08 de	242.67 g	302.00 cd	15.73 d	17.94 cd	2.64 a	2.58 de
	N450	165.41 ab	163.58 ab	69.23 ab	73.09 b	327.67 b	342.67 ab	20.27 b	22.37 b	2.59 ab	2.95 abcd
***F*-values and significance level**											
Biochar		3.74*	12.71***	3.89*	9.41***	18.77***	10.32***	5.93**	7.08***	1.98 ns	0.97 ns
Nitrogen		313.88***	534.63***	206.01***	430.99***	1003.43***	971.36***	270.97***	481.41***	1.42 ns	5.60**
Biochar × Nitrogen		2.18 ns	7.79***	3.50*	11.24***	8.57***	4.03*	5.38**	8.14***	2.12 ns	0.21 ns

B0, 0 t ha^–1^ biochar; B15, 15 t ha^–1^ biochar; B30, 30 t ha^–1^ biochar; B60, 60 t ha^–1^ biochar; N0, 0 kg ha^–1^ nitrogen; N150, 150 kg ha^–1^ nitrogen; N300, 300 kg ha^–1^ nitrogen, and N450, 450 kg ha^–1^ nitrogen. Different letters within the columns show a significant difference between the treatments at p = 0.05 according to the LSD test, and asterisks represent a significant difference at *p < 0.05, **p < 0.01, and ***p < 0.001 level; ns, not-significant.

The integration of biochar and nitrogen fertilizer caused significant variations in seed quality traits of rapeseed in both years (Table 7). In comparison to B0 + N0, the combination of B15 + N225 increased the protein content by 19% in the first year and 13% by B30 + N450 treatment in the second year. Similarly, the combined application of biochar at 30 t ha^–1^ and nitrogen at 225 kg ha^–1^ increased oil content by 7 and 10% during the first year and second year, respectively, as compared to the B0 + N0 treatment (Table 7). Although integration of biochar and nitrogen did not significantly improve other seed quality indices, biochar integration with nitrogen produced higher values by increasing linolenic acid up to 2% in B15 + N225 and 6% in B30 + N75 compared to B0 + N0 during the first and second year, respectively. Likewise, the values of linoleic acid were increased by 8% in B30 + N225 and 5% in B15 + N450 compared to B0 + N0 treatment, respectively, in the first and second year (Table 7). The growth, yield, and quality traits suggest that coupling biochar and nitrogen is more effective and beneficial than a single application of nitrogen fertilizers.

**TABLE 7:** Integrated effect of biochar and nitrogen application on rapeseed seed quality traits during 2019–2020 and 2020–2021.

Biochar	Nitrogen	Oil content (%)		Protein content (%)		Linoleic (%)		Linolenic (%)		Palmitic (%)	
		2019–2020	2020–2021	2019–2020	2020–2021	2019–2020	2020–2021	2019–2020	2020–2021	2019–2020	2020–2021
B0	N0	42.75 b	41.36 de	18.34 de	20.95 cd	16.22 a	16.57 a	7.24 a	7.00 ab	4.68 ab	4.29 abc
	N75	43.90 ab	41.92 cde	19.46 bcde	20.34 d	16.64 a	16.54 a	7.09 a	7.29 ab	4.73 ab	4.25 abc
	N225	43.85 ab	44.45 abcd	20.09 abcd	22.88 abc	17.27 a	17.19 a	7.30 a	7.36 ab	4.66 ab	4.15 abc
	N450	42.67 b	40.46 e	18.57 cde	21.34 cd	16.74 a	17.01 a	7.24 a	6.95 ab	4.66 ab	4.11 bc
B15	N0	44.08 ab	42.98 abcde	19.29 bcde	21.71 abcd	16.45 a	17.10 a	7.53 a	6.89 ab	4.54 ab	4.15 abc
	N75	45.02 a	43.70 abcde	19.68 abcde	22.78 abc	16.78 a	16.28 a	7.15 a	7.21 ab	4.71 ab	4.14 abc
	N225	44.82 ab	44.76 abc	21.98 a	23.62 ab	16.89 a	16.27 a	7.39 a	7.20 ab	4.66 ab	4.07 c
	N450	45.23 a	42.95 abcde	20.92 abc	23.07 abc	17.16 a	17.53 a	7.33 a	7.05 ab	4.73 ab	4.28 abc
B30	N0	43.60 ab	42.13 cde	19.11 bcde	21.42 bcd	16.88 a	16.98 a	7.29 a	7.30 a	4.76 ab	4.24 abc
	N75	44.39 ab	43.73 abcd	19.38 bcde	21.25 cd	17.09 a	17.28 a	7.36 a	7.44 ab	4.54 ab	4.39 ab
	N225	45.76 a	45.62 a	21.58 ab	23.76 a	17.55 a	16.83 a	7.30 a	7.29 ab	4.86 a	4.34 abc
	N450	44.71 ab	43.82 abcd	20.11 abcd	23.86 a	16.85 a	17.51 a	7.34 a	7.19 ab	4.36 b	4.25 abc
B60	N0	45.34 a	42.29 bcde	17.80 de	21.42 bcd	16.70 a	16.95 a	7.25 a	6.82 ab	4.60 ab	4.43 a
	N75	44.65 ab	45.50 cde	17.24 de	22.65 abc	16.41 a	16.66 a	7.35 a	7.19 ab	4.74 ab	4.21 abc
	N225	44.82 ab	45.42 ab	18.19 e	21.30 cd	16.42 a	17.23 a	7.34 a	6.69 b	4.70 ab	4.25 abc
	N450	45.49 a	44.90 abc	19.60 abcde	22.81 abc	16.92 a	16.04 a	7.38 a	7.28 ab	4.61 ab	4.28 abc
***F*-values and significance level**											
Biochar		4.06*	3.43*	5.36*	3.47*	0.69 ns	0.51 ns	0.33 ns	1.16 ns	0.08 ns	1.74 ns
Nitrogen		0.86 ns	4.62*	3.62*	3.82*	0.71 ns	0.26 ns	0.18 ns	0.92 ns	0.51 ns	0.38 ns
Biochar × Nitrogen		0.62 ns	0.55 ns	0.72 ns	1.29 ns	0.36 ns	0.92 ns	0.22 ns	0.58 ns	0.67 ns	0.73 ns

B0, 0 t ha^–1^ biochar; B15, 15 t ha^–1^ biochar; B30, 30 t ha^–1^ biochar; B60, 60 t ha^–1^ biochar; N0, 0 kg ha^–1^ nitrogen; N150, 150 kg ha^–1^ nitrogen; N300, 300 kg ha^–1^ nitrogen, and N450, 450 kg ha^–1^ nitrogen. Different letters within the columns show a significant difference between the treatments at p = 0.05 according to the LSD test, and asterisks represent a significant difference at *p < 0.05, **p < 0.01, and ***p < 0.001 level; ns, not-significant.

### Correlation Coefficients and Principal Component Analysis of Soil Physiochemical Properties, Enzymes, and Yield

**FIGURE 7:**
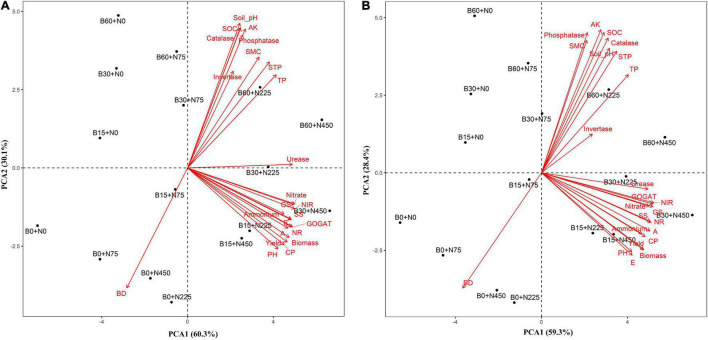
Principal component analysis of soil physiochemical properties, growth and yield traits in 2019–2020 **(A)** and 2020–2021 **(B)**. SOC: soil organic carbon, TP: total phosphorus, AK: available potassium, STP: soil total porosity, SMC: soil moisture content, BD: bulk density, NR: nitrate reductase, NIR: nitrite reductase, GS: glutamate synthase, GOGAT: glutamine synthetase, A: photosynthesis, E: transpiration, SS: soluble sugar, CP: crude protein, PH: plant height.

The mutual relationship between the observed indices in 2 years was evaluated by performing Pearson correlation analysis (Figures 7A,B). The correlation of soil pH and SOC was positive with TP, AK, soil total porosity, soil moisture content, phosphatase, and catalase; however, the correlation of soil bulk density was negative with other parameters in both years. Soil nitrate-nitrogen, ammonium nitrogen, and urease had a strong positive correlation with the enzymatic activities of NR, NIR, GS, GOGAT, and plant growth and yield traits, such as photosynthesis, transpiration, soluble sugar, crude protein, plant height, biomass, and yield in both years (Figures 7A,B).

**FIGURE 8:**
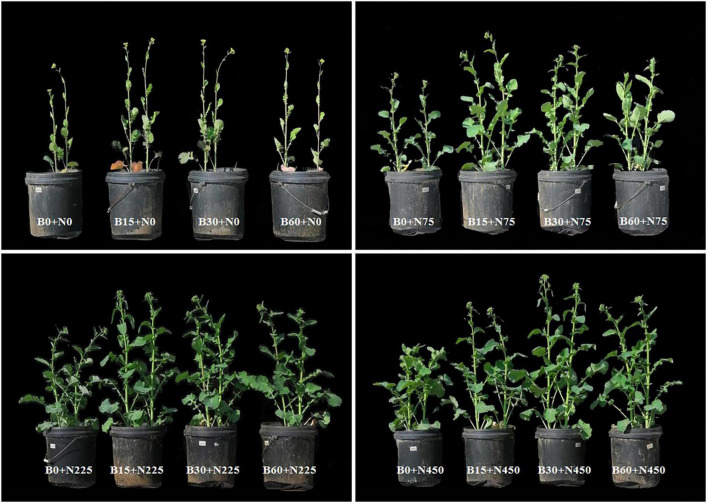
Rapeseed phenotypes representing each treatment combination of biochar and nitrogen.

Multivariate analysis of PCA showed obvious variations between soil physiochemical properties and enzymatic activities with plant physiological and yield traits during 2 years in the first and second principle component (Figures 8A,B). The total variance during the first year in two principal components was 90% consisting of variance of 62 and 26% explained by PCA1 and PCA2, respectively, while a total variance of 87% was counted during the second year, among which the PCA1 explained 59% and PCA2 explained 28% of the variance in individual traits of soil and plant.

## Discussion

A single application of high nitrogen fertilizer could reduce nitrogen use efficiency, increase soil acidity and adversely affect rapeseed growth. However, biochar nutrient retention ability can compensate for the high nitrogen toxicity due to the highly porous structure and liming effect (Khan et al., 2020, 2021b). In the current study, the biochar treated soil exhibited significant variations in soil nutrient availability and uptake compared to soil without biochar. In both years, the biochar addition significantly improved SOC, soil pH, total porosity, bulk density, and soil moisture content compared to untreated control soil (B0 + N0) (Tables 2, 3).

Soil organic carbon promotes soil quality by improving soil’s biological and physiochemical properties (Khan et al., 2020), and the stabilization of SOC can be negatively affected by decreasing soil pH (Sheng et al., 2016). The improvement in soil SOC could be related to the enormous carbon content in rice straw biochar which might be released to the soil as a labile carbon. Furthermore, as our findings revealed, the increments in soil total porosity, moisture content, and decrease in bulk density of soil can provide more space and water to facilitate the habitat of microorganisms, and thus, microorganisms can increase the accumulation of soil organic carbon. The increment in soil pH in this study could be attributed to the high ash content released by biochar, as the applied rice straw biochar contained 19.43% ash content. Previously, it has been observed that alkaline bases on biochar particles released high cations to soil and improved the protons consumption reactions that reduced soil acidity and increased soil pH (Schulz et al., 2013). Similar profound results of soil physiochemical properties upon biochar addition have been reported previously (Lawlor, 2002; Kameyama et al., 2014).

Biochar is an organic reservoir of nitrogen, phosphorus, and potassium that can elevate the concentrations of vital essential nutrients in the soil (Khan et al., 2021b). The present study showed that biochar addition significantly increased NO${}_{3}^{-}$, NH${}_{4}^{+}$, TP, and AK compared to pots with no biochar; however, the biochar application at 60 t ha^–1^ decreased NO${}_{3}^{-}$ and NH${}_{4}^{+}$ compared to 30 t ha^–1^ (Tables 2, 3). These positive results of essential nutrients might be due to the pronounced effects of biochar on soil organic carbon and the physical properties of soil that improved the soil retention capacity to prevent the nutrients leaching and increased the availability of these essential nutrients in biochar treated soil. The current results of our study can be correlated with increased cation and anion exchange capacities under biochar addition (Mavi et al., 2018), which caused a decrease in nutrient loss from soil and increased retention and concentrations of NO${}_{3}^{-}$-N and NH${}_{4}^{+}$-N (Abbruzzini et al., 2019). The biochar amendment can also cause negative and adverse effects on soil nutrients availability due to the high *C*:*N* ratio of biochar which can increase immobilization of nitrogen in the soil and decrease the availability of N for plant uptake (Lehmann and Joseph, 2012).

During 2 years of study, the soil enzymatic activities of urease, soil catalase, alkaline phosphatase, and invertase were significantly increased under biochar application compared to pots with no biochar (Figure 1). Soil enzymes play an essential role in various soil biochemical processes such as SOM mineralization and other nutrient cycles (Makoi and Ndakidemi, 2008). In the present study, the biochar effect was significant on all enzymes; in contrast, the nitrogen effect was only effective on urease and negatively affected the activities of other enzymes, which indicates the positive and synergetic effect of biochar in promoting soil quality. The positive response of urease to biochar addition is crucial as this enzyme takes part in cycling and availability of N (Harter et al., 2014), and this high activity of urease in the presence of biochar might be due to the high N mineralization and retention effect of biochar. These results suggest that biochar can more efficiently promote the hydrolysis of applied nitrogen fertilizers.

Phosphatase activity is related to soil factors such as soil pH and soil moisture (Gong et al., 2018). Thus, the increased phosphatase activity is likely to be correlated to the porous structure, ash content, and high organic carbon in biochar that resulted in an increased soil pH and soil moisture content upon biochar addition in our present study. The activity of microbial oxidative reductase, which is driven by soil catalase, is related to the metabolism of soil microbes’ oxidative activities (Weaver et al., 1994). The possible mechanism for incrementing catalase activity in the present study is that biochar application benefited the oxidative activities of soil microbes by increasing soil porosity and moisture content to promote the habitat of soil microbes. The activity of soil invertase depends on organic carbon, mineral N, and P in soil (Zhang et al., 2005). In our findings, the elevated activity of invertase might be due to high soil organic carbon and enhanced mineralization of nitrogen and phosphorus with biochar addition. Furthermore, the decreased values of urease and invertase in 60 t ha^–1^ biochar amended pots might be due to high soil pH and adsorption effect that reduced their activities (Elzobair et al., 2016). Similar positive results of soil enzymes with biochar addition have been reported previously (Oladele, 2019). Altogether, these high enzymatic activities under biochar amendment reflect biochar’s positive role on soil quality and nutrients cycling.

The plant N content, TNA, NUE, and activities of these nitrogen-related enzymes were higher in the combined application of biochar and nitrogen compared to control or a single application of high nitrogen fertilizer (Figures 2, 3). Plant N uptake is determined by soil available N content, and biochar application significantly increased the contents of NO${}_{3}^{-}$-N and NH${}_{4}^{+}$-N in both years of this study. This increase can be related to a certain amount of N present in biochar and elevated urease activity under biochar addition, which helped in the hydrolysis of mineral N availability and thus increased the plant N content and activities of nitrogen-related enzymes. The lower activities of N-related enzymes in high biochar application at 60 t ha^–1^ indicate reduced N availability for plant uptake and conversion due to high sorption of N to biochar surface (Jones et al., 2012). The optimized availability and uptake of inorganic N by a plant that elevated the activities of NR, NIR, GS, and GOGAT can be related to single and aromatic carbon rings in biochar that increased the nutrients retention capability of biochar (Baronti et al., 2014). The biochar particles also exhibit energy substances that directly adsorb applied nitrogen as a microbial substrate to increase total nitrogen accumulation and nitrogen use efficiency (Lehmann, 2007).

Plant soluble sugar, a final product of photosynthesis, is the main crucial physiological process that governs plant carbon assimilation and reflects a plant’s growth status. In this study, compared to the sole application of nitrogen fertilizer, we found a significant effect of biochar on physiological traits of rapeseed in both years (Table 5), that subsequently increased the contents of soluble sugar and crud protein (Figure 5). Besides the effect of stomatal and non-stomatal traits in photosynthesis, plant essential nutrients also play a vital role in the physiological processes of plants (Lyu et al., 2016). The increasing trend in leaf photosynthetic rates, soluble sugar, and protein content is associated with optimum soil nutrients availability, plant uptake, and use efficiency. This optimum nutrients supply can be attributed to the optimized results of soil and plant enzymes obtained in this study with biochar addition are responsible for the improved mineralization, transportation, and assimilation of plant nutrients. Moreover, the porous structure of biochar can absorb more water particles in the soil to increase water holding capacity (WHC) and roots proliferation to enhance the leaf turgor pressure and photosynthesis. Similar ameliorative effects of biochar on plant physiological status have been reported previously (Xu et al., 2015; Khan et al., 2021a). However, contrasting results also explain that nutrient was not the limiting factor for photosynthetic rates (Akhtar et al., 2014) or applied biochar was less nutritious (Alburquerque et al., 2014).

Previous studies showed that soil fertility and plant nutritional management strategies significantly influence crop growth and yield attributes (Xu et al., 2015; Fang et al., 2021). In both years of this study, we observed that a single application of high nitrogen and high biochar negatively affected the rapeseed plant growth (Table 6 and Figure 6) and physiological status, however; the application of biochar in combination with nitrogen significantly elevated the growth, yield components, and seed quality traits in biochar amended pots than those without biochar (Tables 6, 7). The increasing trend in rapeseed growth and yield was positive up to 30 t ha^–1^ biochar and 225 kg ha^–1^ nitrogen application, while a decreasing trend was observed at 60 t ha^–1^ biochar and 450 kg ha^–1^ nitrogen, which indicated the adversity of high amendment levels. The declined growth with short-statured plants (Figure 6) suggests that a single high nitrogen application has increased the soil acidity and decreased the availability and uptake of other essential nutrients. The excessive application of single nitrogen fertilizers decreased the nitrogen use efficiency and grain yield, which could be attributed to the loss of extra nitrogen from the soil and the acidity of high nitrogen fertilizer (Shi et al., 2012). On the other hand, high biochar application can also exhibit certain uncertainties that negatively alter the soil ecosystem, nutrients dynamics, and plant growth. The possible mechanism for this could be the high binding forces in soil with high biochar concentration that decreased the availability of the nutrients and increased the salt concentration due to its liming effect. The lower values of NO${}_{3}^{-}$-N and NH${}_{4}^{+}$-N under high biochar 60 t ha^–1^ might have reduced plant growth be due to negative and adverse effects of biochar on soil nutrients availability due to the high *C*:*N* ratio of biochar which can reduce the N availability to plant by elevating immobilization of N in the soil (Lehmann and Joseph, 2012). High application of biochar can reduce the population of beneficial mycorrhizal fungi due to changes in soil pH and toxic effects of salts contents present in biochar (Birk et al., 2009; Mukherjee and Lal, 2014). The presence of heavy metals and salts in biochar induce toxic effects on soil pH and reduce the activities of beneficial microbes (Lehmann and Joseph, 2012).

The combined application of biochar and nitrogen fertilizer improved rapeseed growth, yield, and quality traits which can be related to the presence of microbial biomass, and the porous surface of biochar can absorb a large amount of nitrogen by increasing immobilization to decrease the adversity of high nitrogen fertilizer. Similar results were reported previously (Khan et al., 2020) that biochar absorption ability reduces the nitrogen toxicity in the early plant growth stage and then enhances the nitrogen availability in the middle and late stages of plant growth to meet the plant nitrogen requirements. Furthermore, the biochar also provides nitrogen, potassium, and phosphorus to the soil in bioavailable forms, which might have promoted plants’ nutrient supply and uptake to elevate the rapeseed growth and yield (Laird et al., 2010). The positive and significant results of rapeseed growth, yield, and quality traits under combined application of biochar and nitrogen suggest that biochar can ameliorate the nutrients toxicity and promote the soil quality and, consequently, the sustainable production of rapeseed.

The relationship and significant differences of different biochar and nitrogen fertilizer levels were evaluated among soil (physiochemical properties and enzymatic activities) and plant (morpho-physiological and yield) traits by performing PCA analysis (Figures 8A,B). A strong and positive correlation was observed between plant height, biomass, photosynthesis, transpiration, soluble sugar, soluble protein, nitrogen-assimilation enzymes, and grain yield. The treatments (B15 + N225, B15 + N450, B30 + N225, and B30 + N450) were existed in the same quarter and separated from other treatments, representing the significant effect of combined application of low biochar levels with low and high nitrogen levels on plant growth attributes (Figures 8A,B). similarly, the treatments (B60 + N225 and B60 + N450) were significantly separated from other treatments by performing a large assembly of positively correlated indices of soil, including soil pH, total phosphorus, available potassium, soil total porosity, soil moisture content, soil organic carbon, phosphatase, invertase, and catalase. In contrast, the soil bulk density was negatively correlated in both years and existed in a separate quarter of the treatments having zero biochar (Figures 8A,B). A mutual relationship among soil and plant attributes under the combined application of biochar and nitrogen levels in both years was evaluated by Pearson correlation analysis (Figures 7A,B). The correlation was found very strong and positive among various soil and plant growth parameters; however, soil bulk density was negatively correlated with all other parameters. The findings of these positive correlation coefficients among soil physiochemical properties, enzymatic activities, and plant growth attributes indicate that biochar amendment had improved soil conditions, nitrogen fertilizer utilization, and plant growth.

## Conclusion

The role of rice straw biochar addition on soil conditions and rapeseed growth under inorganic nitrogen fertilizer was evaluated for 2 years. High or lower nitrogen fertilizer application significantly reduced soil properties, soil and plant enzymatic activities, nitrogen use efficiency, and growth and yield of rapeseed. However, the combined amendment of biochar with nitrogen alleviated the adverse effect of high or lower nitrogen fertilizer application on soil conditions and rapeseed growth. Under different treatments combination, amendment of biochar at 30 t ha^–1^ with nitrogen 450 kg ha^–1^ resulted in high soil nitrogen availability (NO${}_{3}^{-}$-N and NH${}_{4}^{+}$-N), improved physiological growth (photosynthesis, transpiration, soluble sugar, and crude protein), increased enzymatic activities (Urease, NR, NIR, GS, and GOGAT) and prominent growth and yield (plant height, biomass, pods plant^–1^ and grain yield). Applying biochar at 15 t ha^–1^ can also offset the deficiency of lower nitrogen as B15 + N225 resulted in the highest NUE of 29% in both years. In conclusion, a proper combination of biochar and nitrogen is recommended due to the positive effects of biochar on soil status and rapeseed productivity and quality traits and mitigating the adverse effects of high nitrogen toxicity or lower nitrogen deficiency.

## Data Availability Statement

The original contributions presented in the study are included in the article/supplementary material, further inquiries can be directed to the corresponding authors.

## Author Contributions

LH, LL, and ZK designed the research. ZK, MK, and KaZ performed the experiments and collected data. JB and KuZ revised the manuscript and helped in manuscript revision. ZK wrote the manuscript. All authors contributed to the article and approved the submitted version.

## Conflict of Interest

The authors declare that the research was conducted in the absence of any commercial or financial relationships that could be construed as a potential conflict of interest.

## Publisher’s Note

All claims expressed in this article are solely those of the authors and do not necessarily represent those of their affiliated organizations, or those of the publisher, the editors and the reviewers. Any product that may be evaluated in this article, or claim that may be made by its manufacturer, is not guaranteed or endorsed by the publisher.
